# In vitro evaluation of three engineered multispecies endodontic biofilms on a dentinal disk substrate

**DOI:** 10.1080/26415275.2023.2281091

**Published:** 2023-12-21

**Authors:** Wajih Hage, Dolla Karam Sarkis, Mireille Kallasy, May Mallah, Carla Zogheib

**Affiliations:** a Department of Endodontics, Universite Saint-Joseph, Beirut, Lebanon; b Department of Bacteriology, Universite Saint-Joseph, Beirut, Lebanon; c Department of Chemistry, Universite Saint-Joseph, Beirut, Lebanon

**Keywords:** Biofilm, endodontic biofilm, infection

## Abstract

The aim of this study was the development of a complex multispecies endodontic biofilm using *Candida albicans*, *Proteus mirabilis* and *Pseudomonas aeruginosa* on a biofilm of *Enterococcus faecalis* in a dentinal substrate design.

The endodontic pathology is a biofilm-mediated infection, and the aim of root canal therapy is to reduce, as much as possible, the bacterial population. Thus, it is important to develop a laboratory endodontic biofilm to test the effect of new irrigation and obturation techniques on reduction of bacterial count.

The culture of *Enterococcus faecalis* from ATCC 29212 began with aerobic cultivation on blood agar, followed by transfer to Brain Heart Infusion (BHI) broth with 5% sucrose. Incubation occurred in a shaker at 37 °C for 24 h, followed by an additional 24-h static phase. After 10 d, *Proteus mirabilis*, *Pseudomonas aeruginosa*, and Candida albicans were introduced sequentially in three distinct groups. Group 1: the order of addition was *Candida albicans*, *Proteus mirabilis*, and *Pseudomonas aeruginosa*; Group 2: the order was *Pseudomonas aeruginosa*, *Candida albicans*, and *Proteus mirabilis*; and Group 3: *Proteus mirabilis*, *Pseudomonas aeruginosa*, and *Candida albicans.* After 16 days, the biofilm was carefully extracted, transferred to sterile BHI, and dissected using a sterile needle technique. Subsequently, an optical density test, bacterial counts, and colony enumeration were performed on various agar plates.

Group 2 in which *Pseudomonas aeruginosa* was added directly after *Enterococcus faecalis* followed *by Candida albicans* and *Proteus mirabilis* showed significantly greater total bacterial count than the other two groups.

## Introduction

Endodontic pathology is a biofilm-mediated infection, and the removal of bacterial biofilm from the root canal system is the primary goal of treatment [1]. The endodontic infection is caused by germs that develop on the surface of the tooth [2]. It is critical to apply the biofilm idea to endodontic microbiology in order to better understand the pathogenic potential of the root canal microbiota and to lay the groundwork for innovative disinfection methods [3]. The first step is to comprehend how root canal bacteria create a biofilm that resists endodontic treatment. The biofilm community not only protects bacteria from the host’s defense system, but it also makes the bacteria more resistant to a number of disinfectants used in root canal irrigation techniques and infection therapy [4]. Biofilm removal and effective biofilm bacteria killing are required for successful therapy of many disorders [5].

The root canal system, on the other hand, has an extremely complicated anatomy, with isthmuses, lateral extensions, apical deltas, lateral canals, and dentinal tubules providing protection to microorganisms from instruments and disinfectants [6]. Furthermore, the bacteria’s biofilm lifestyle in the root canal adds to the difficulty [7]. The microbial cells are immersed in a self-produced extracellular matrix and adhere to the canal walls. Biofilm cells are far more resistant to most antimicrobials and host defenses than their planktonic cousins [3, 5].

Despite the fact that laboratory models are always a simplification of the infected root canal’s clinical reality, they are nonetheless useful tools for evaluating the preliminary effectiveness of novel or alternative root canal disinfection techniques [8]. As human dentine is the biofilm’s natural habitat, using it as a substrate for biofilm growth is the most logical choice [9]. The bacterial attachment to a surface and permanent adhesion to the substrate are the primary events in the creation of a biofilm. Bacterial adhesion to dentine is more likely to occur on the protein-rich region of the dentine matrix rather than the mineral component [10].

The aim of this study was the development of a complex multispecies endodontic biofilm using *Enterococcus faecalis*, *Proteus mirabilis, Pseudomonas aeruginosa and Candida albicans* on a dentinal substrate design in order to test the efficiency of future irrigation and obturation techniques on the reduction of the bacterial count.

## Methodology

The study protocol was approved by the “Ethics Committee” of the Saint Joseph University (FMD200).

### Dentinal disk preparation

Twenty freshly extracted, caries-free molars had their outer surfaces cleaned with sodium hypochlorite (NaOCl) 5% before being rinsed with sterile water. In the following step, teeth were inserted into plastic molds (vinyl polysiloxane impression material; Dentsply DeTrey GmbH, Konstanz, Germany) and secured to the base using putty. EpoxyResin® base and hardener were mixed in a 5:1 ratio (Buehler, Lake Bluff, IL USA). The resin was vigorously mixed to obtain a consistent consistency and to let any trapped air bubbles escape. The prepared resin was poured into the plastic mold gradually until the tooth was entirely enclosed. At room temperature, the resin was left to cure for 24 h. The resin was then removed from the plastic mold. Using an Isomet®2000 precision saw, resin samples were cut into 2 mm wafers, producing a total of 30 disks (three disks for each group). After cutting, the disks were polished using TEXMET® polishing disks with Metadi® (Buehler, Lake Bluff, IL USA). Each disk was rinsed in water. The smear layer and any potential contaminants were removed from the disk surfaces for 1 min for each solution using a small sticky brush and NaOCl 5% and 17% Ethylenediaminetetraacetic EDTA solution (ACTEON, Merignac, France). To ensure complete solvent removal, disks were rinsed under running water for 5 h. All samples were then autoclaved for 20 min at 120 °C. Dentinal disks were stored in sterile water at 4 °C before use. To standardize the sample the chosen diameter of the dentinal disk selected was 5mm.

### Monobacterial biofilm formation

*Enterococcus faecalis* from ATCC 29212 was obtained from Saint Joseph University’s Microbiologic Department and grown aerobically on blood agar for 48 h at 35 °C. Then, colonies were raised in Brain Heart Infusion (BHI) (Becton Dickinson, Sparks, MD, USA) broth with 5% saccarose at 37 °C for 24 h in a shaker incubator. Agitation was applied to ensure uniform growth of the biofilm and to prevent mass transfer limitations, mimic natural conditions, achieve higher growth rates, conduct stress testing, and maintain biofilm homogeneity [1, 3], and was followed by 24 h in a static environment. A sterile BHI broth inoculum was made with 5% saccarose and turbidity was set to 0.5 McFarland, or roughly 1.5 10^8^ colony forming units per milliliter (CFU/mL). A sample of 27 dentinal disks that had previously been treated with collagen type 1 were immediately placed in 10 µl of the culture in sterile cups, and cultured at 37 °C for 14 d. Collagen type 1 was added because it provides a biologically relevant substrate that mimics the extracellular matrix of human tissues, promoting bacterial adhesion and biofilm development) [1] sterile BHI broth + 5% saccarose was added every 24 h to maintain the viability of the culture.

Three different species, *Proteus mirabilis, Pseudomonas aeruginosa and Candida albicans*, were selected and cultivated overnight in BHI at 37 °C in a shaker incubator with a shaking speed of 100 rpm for 24 h. The disks were then placed for 24 h in static incubation at 37 °C. The aim of this procedure was to promote uniform growth by ensuring a steady supply of nutrients and oxygen, to prevent the formation of stagnant boundary layers, facilitating efficient nutrient diffusion and waste removal, to accelerate microbial growth rates, enabling faster biofilm production for experimental purposes, and finally to contribute to biofilm homogeneity, ensuring research reliability and reproducibility). Inoculum was prepared in sterile BHI broth and turbidity was set to 0.5 McFarland corresponding to approximately 1.5 × 10^8^ CFU/mL. Ten µl of the culture was immediately inoculated on dentinal disks in polystyrene microtitre plates for 10 d.

The biofilm development process involved two distinct phases: aerobic and anaerobic incubation. In the aerobic phase, initial growth was facilitated by placing samples in sterile petri dishes covered with breathable lids to allow oxygen exchange. Following this, a transition was made to an anaerobic environment using a specialized anaerobic chamber. This chamber was filled with a gas mixture of 5% CO_2_, 10% H_2_, and 85% N_2_ to displace any remaining oxygen after thorough flushing. The anaerobic phase was carefully regulated at 37°C for 48 h. The transition between aerobic and anaerobic conditions was managed delicately to minimize stress on the developing biofilm. This was achieved by gradually introducing the samples to the anaerobic chamber. Additionally, a controlled release mechanism was employed to slowly adjust the gas composition within the chamber, allowing the biofilm to acclimate to the changing conditions.

Three cultures were used as a negative control. Each culture’s supernatant was discarded, and plates were then cleaned three times with PBS to get rid of any loose cells. Plates were then fixed with methanol for 30 min., stained with 1% crystal violet (CV) for 30 min., and rinsed with distilled water to make sure the monospecies biofilm had actually formed.

The dentinal disks were randomly divided into three groups as follows.

Group 1: 10 µl of the *Candida albicans* culture were added to the microtitre plates at day 10, 10 µl of the *Proteus mirabilis* culture were added at day 14, and 10 µl of the *Pseudomonas aeruginosa* culture were added at day 16.

Group 2: 10 µl of the *Pseudomonas aeruginosa* culture were added to the microtitre plates at day 10, 10 µl of the *Candida albicans* culture were added at day 14, and 10 µl *Proteus mirabilis* of the culture were added at day 16.

Group 3: 10 µl of the *Proteus mirabilis* the culture were added to the microtitre plates at day 10, 10 µl of the *Pseudomonas aeruginosa* culture were added at day 14, and 10 µl of the *Candida albicans* culture were added at day 16.

These timeframes were chosen to align with the typical growth and colonization patterns of the specific microorganisms used in this study [1].

The multispecies biofilm was then removed using forceps and placed in sterile BHI broth for 15 min after vortex. The biofilm was dissected using the sterile needle technique for 15 min. Then an optic density test was realized, followed by gram staining and a microscopic observation. Fifty µl of the liquid medium was serially diluted in sterile BHI broth and plated on different agars (YCG for C., Cetrimid Agar for *Pseudomonas aeruginosa*, slantez and bartley for *Enterococcus faecalis*, uriselect for *Proteus mirabilis*). Culture media was placed at 37 °C for 48 h. Colonies were counted and confirmed by colony morphology observation on the agar of choice.

### Statistical analysis

Data were analyzed using IBM SPSS Statistics for Windows, version 26 (IBM Corp., Armonk, NY, USA). Descriptive statistics of the quantitative variables were summarized and presented as medians (1^st^ and 3^rd^ quartiles), means ± standard deviations, and minimum and maximum values. Normality of distribution of the quantitative variables were assessed using the Shapiro-Wilk test. The one-way analysis of variance (ANOVA) was used to compare values between groups when normality of distribution was assumed, while the Kruskal-Wallis test was used when normality of distribution was not assumed. These tests were followed by the Bonferroni post-hoc test for multiple pairwise comparisons. All tests were two-tailed and the level of significance was set at 5%.

## Results

**Figure 1.**
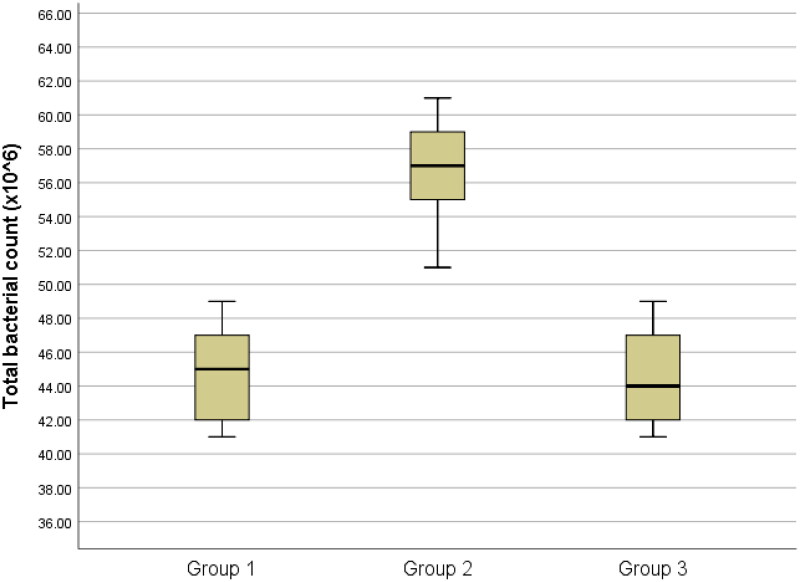
Box-plots of total bacterial count among groups

**Figure 2.**
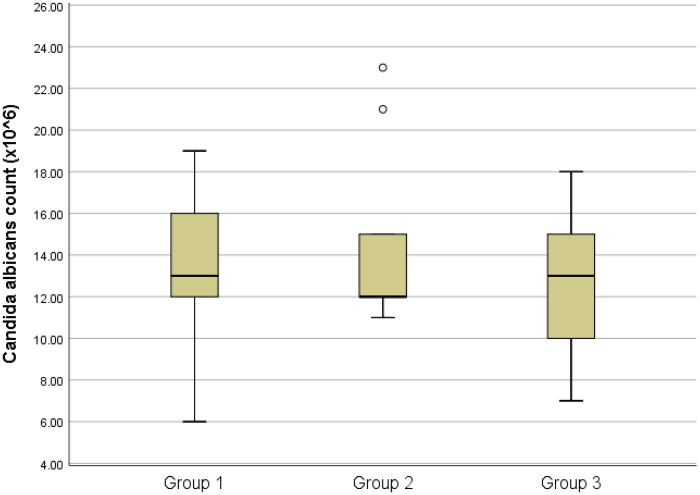
Box-plots of Candida albicans count among groups

**Figure 3.**
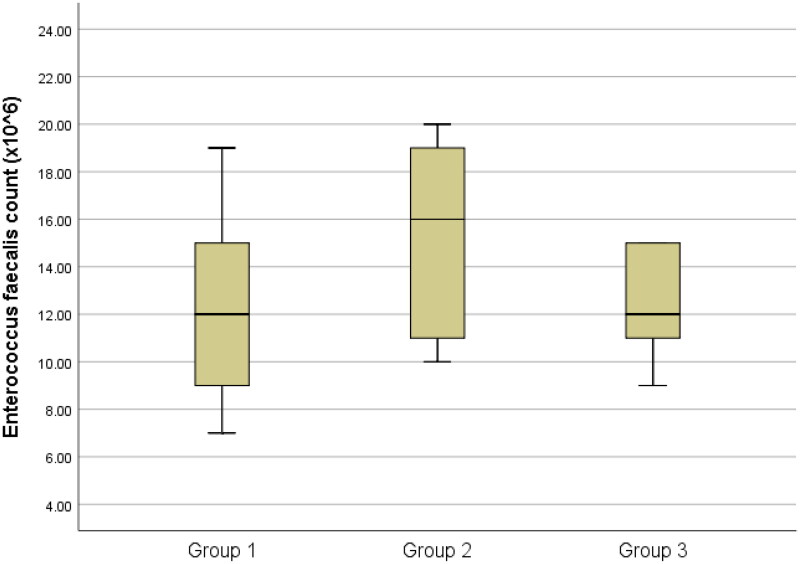
Box-plots of Enterococcus faecalis count among groups

**Figure 4.**
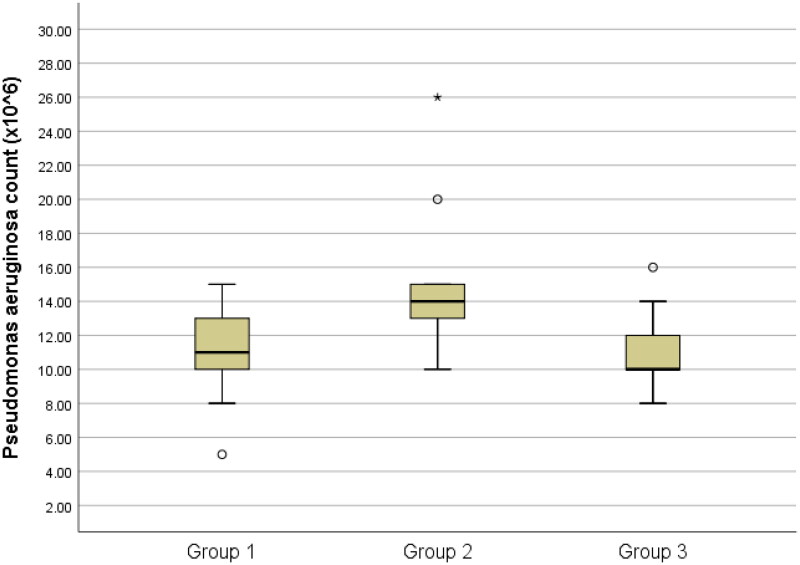
Box-plots of Pseudomonas aeruginosa count among groups

**Figure 5.**
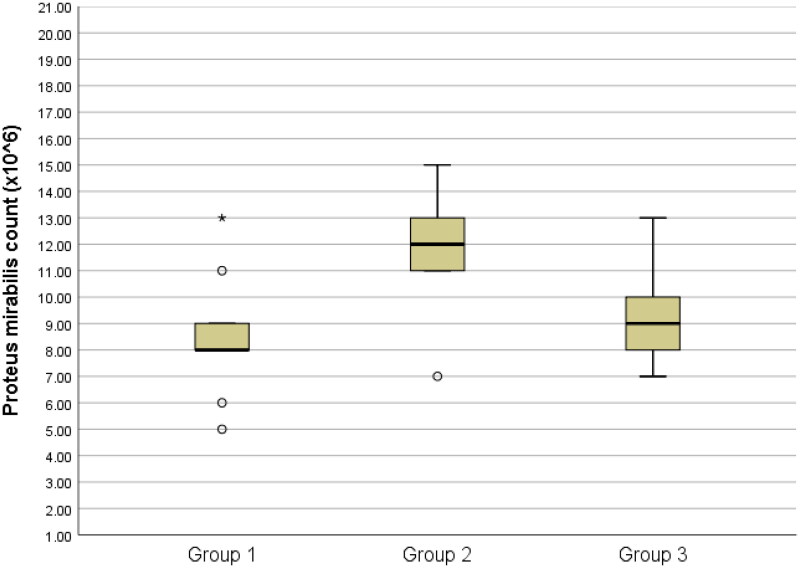
Box-plots of Proteus mirabilis count among groups

**Figure 6.**
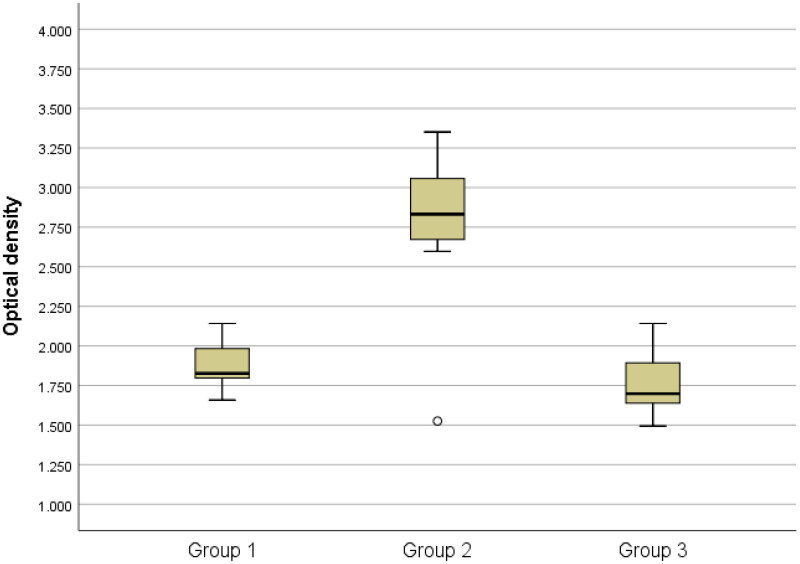
Box-plots of optical density among groups

The results of the total bacteria counts are presented in Table 1 and Figs. 1-5., while the optical density results are presented in Table 2 and Fig. 6.

**Table 1.** Descriptive statistics of bacterial count (x10^6^) and comparisons between groups

	Group 1 (n = 9)	Group 2 (n = 9)	Group 3 (n = 9)	*p*-value	Total (N = 27)
Total bacterial count (x10^6^)					
*Mean ± SD*	44.78 ± 2.91^B^	56.44 ± 3.47^A^	44.55 ± 3.05^B^		48.59 ± 6.42
*Median (Q1 – Q3)*	45 (42 – 47.5)^B^	57 (53.5 – 59.5)^A^	44 (41.5 – 47.5)^B^	<0.001*	47 (43 – 55)
*Minimum*	41	51	41		41
*Maximum*	49	61	49		61
*Candida albicans* (x10^6^)					
*Mean ± SD*	13.11 ± 4.34^A^	14.33 ± 4.53^A^	13.22 ± 3.73^A^		13.55 ± 4.09
*Median (Q1 – Q3)*	13 (9.5 – 16.5)	12 (11.5 – 18)	13 (10 – 16.5)	0.795	13 (11 – 16)
*Minimum*	6	11	7		6
*Maximum*	19	23	18		23
*Enterococcus faecalis* (x10^6^)					
*Mean ± SD*	12.44 ± 3.94^A^	15.44 ± 4.19^A^	12.44 ± 2.24^A^		13.44 ± 3.71
*Median (Q1 – Q3)*	12 (8.5 – 15)	16 (10.5 – 19.5)	12 (10.5 – 15)	0.141	13 (10 – 15)
*Minimum*	7	10	9		7
*Maximum*	19	20	15		20
Pseudomonas aeruginosa (x10^6^)					
*Mean ± SD*	10.89 ± 3.10^B^	15.55 ± 4.72^A^	11.22 ± 2.44^B^		12.55 ± 4.04
*Median (Q1 – Q3)*	11 (9 – 13.5)^B^	14 (13 – 17.5)^A^	10 (10 – 13)^B^	0.029*	12 (10 – 14)
*Minimum*	5	10	8		5
*Maximum*	15	26	16		26
Proteus mirabilis (x10^6^)					
*Mean ± SD*	8.55 ± 2.40^B^	11.89 ± 2.31^A^	9.11 ± 1.90^B^		9.85 ± 2.60
*Median (Q1 – Q3)*	8 (7 – 10)^B^	12 (11 – 13.5)^A^	9 (7.5 – 10)^B^	0.009*	8 (10 – 12)
*Minimum*	5	7	7		5
*Maximum*	13	15	13		15

SD = standard deviation; Q1 = first quartile; Q3 = third quartile; *P < 0.05; different uppercase superscript letters indicate statistically significant differences between groups.

**Table 2.** Descriptive statistics of the optical density and comparison between groups

	Group 1 (n = 9)	Group 2 (n = 9)	Group 3 (n = 9)	*p*-value	Total (N = 27)
Optical density					
*Mean ± SD*	1.89 ± 0.16^B^	2.76 ± 0.52^A^	1.78 ± 0.21^B^		2.14 ± 0.55
*Median (Q1 – Q3)*	1.83 (1.78 – 2.05)^B^	2.83 (2.63 – 3.09)^A^	1.70 (1.61 – 1.95)^B^	0.003*	1.91 (1.7 – 2.67)
*Minimum*	1.66	1.52	1.49		1.49
*Maximum*	2.14	3.35	1.95		3.35

SD = standard deviation; Q1 = first quartile; Q3 = third quartile; *P < 0.05; different uppercase superscript letters indicate statistically significant differences between groups.

Group 2 exhibited notably higher total bacterial counts in comparison to both groups 1 and 3, with a statistical significance of P < 0.05. However, there was no statistically significant variation in total bacterial counts between groups 1 and 3 (P > 0.05). (fig.1)

Concerning *Candida albicans* and *Enterococcus faecalis,* there were no statistically significant distinctions observed among the three groups (P > 0.05). (fig.2-3)

In terms of *Pseudomonas aeruginosa* counts, group 2 displayed a significantly higher count compared to groups 1 and 3 (P < 0.05). Nevertheless, there was no statistically significant difference in *Pseudomonas aeruginosa* counts between groups 1 and 3 (P > 0.05). (fig.4)

Similarly, for *Proteus mirabilis* counts, group 2 exhibited a significantly greater count compared to groups 1 and 3 (P < 0.05). Conversely, there was no statistically significant variation in *Proteus mirabilis* counts between groups 1 and 3 (P > 0.05). (fig.5)

Furthermore, a significantly higher optical density was observed in group 2 when compared to groups 1 and 3, with a P-value of <0.05. However, there was no statistically significant difference in optical density between groups 1 and 3 (P > 0.05). (fig.6)

## Discussion

The aim of this study was the development a complex multispecies endodontic biofilm using *Enterococcus faecalis, Proteus mirabilis, Pseudomonas aeruginosa and Candida albicans* on a dentinal substrate design in order to test the efficiency of future irrigation and obturation techniques on the reduction of the bacterial count.

Ideally, typical members of the root canal microbiome are chosen as the constituent species for the endodontic biofilm model [1]. While both Gram-positive and Gram-negative bacteria have species that are regularly found in initial infections [11], Gram-positive species were most frequently used in the literature, because Gram-negative species are less frequently discovered in post-instrumentation or post-medication samples as they are more easily removed compared to Gram-positive species [12]. The use of a Gram-positive species may be justified by the claim that they present the greatest obstacle to eradication [13]. Gram-negative and Gram-positive species should both be present in mixed-species communities because the effects of a particular medication may differ for Gram-negative and Gram-positive bacteria [14]. Regarding the oxygen state facultative anaerobes were chosen because they are responsible for the bulk of persistent infections [15]. However, a combination of strictly and facultative anaerobic species is more relevant in a multispecies model [1, 16]. Working only with anaerobic bacteria, however, entails certain requirements on the experimental workflow, such as the requirement for decreasing media, anaerobic chambers, anaerobic incubation, and maintenance of anaerobiosis throughout the experiments. This complicates the experimental processes in comparison to dealing with facultative anaerobes or aerobic bacteria and calls for specific tools, materials, and staff [16, 17]. *Enterococcus faecalis*, *Proteus mirabilis*, *Pseudomonas aeruginosa*, and *Candida albicans* are four of the most prevalent endodontic biofilm species that we used in our research.

The most often used test organism in endodontic biofilm model systems has been *Enterococcus faecalis* [1, 16, 17]. This species has frequently been isolated from teeth with persistent apical pathosis after root canal therapy [15, 18]. Only two unfriendly conditions that *Enterococcus faecalis* has been known to endure are alkaline surroundings and extended fasting [18]. *Enterococcus faecalis* has the ability to deeply penetrate dentinal tubuli and form biofilms on the root canal wall even when treated with calcium hydroxide [19]. Because of its ability to survive in difficult environmental settings and epidemiological studies that has linked it to post-treatment illness, *Enterococcus faecalis* has long been regarded as an important pathogen in endodontology [20]. This is expected to lead to intensive research examining how well a treatment performs on a monospecies *Enterococcus faecalis* biofilm. However, this tactic deserves critical consideration [1, 17]. The function of *Enterococcus faecalis* as the main reason for endodontic treatment failures is first in question [21]. This is because *Enterococcus faecalis* is not always identified from unsuccessful root fillings that have been investigated, and it is also not usually one of the most prevalent species in the bacterial population [21, 22]. Consequently, *Enterococcus faecalis* is no longer thought to be the main pathogenic species in root canals. Secondly, *Enterococcus faecalis* is a species that develops swiftly under laboratory conditions and is not at all fastidious [23]. Its high isolation frequency and frequent usage as a test species in endodontic biofilm models [22, 24] are likely due to these properties. Hence, the importance of developing a multispecies biofilm.

The fungus most frequently encountered in endodontic root canal infections is *Candida albicans* [25]. Despite being detected by tooth pulp and periradicular tissue cells that trigger immune responses [25, 26], *Candida albicans* manages to elude host defenses and induce cell death. To withstand intracanal cleaning agents and endodontic therapies, *Candida albicans* adheres to tooth dentine, forms biofilms, and infiltrates dentinal tubules [27]. The resilience of *Candida albicans* to the majority of conventional medications allows it to persist within biofilms and intratubular dentine [26]. Consequently, *Candida albicans* has been associated with cases of stubborn or persistent root canal infections [26, 27].

In this experiment, *Pseudomonas aeruginosa* and *Proteus mirabilis* were chosen due to their propensity to produce biofilms and significant bioluminescence activity [15, 28]. *Pseudomonas aeruginosa* and *Proteus mirabilis* morphology (Gram-negative rods ranging in length from two to three millimeters) is remarkably comparable to other Gram-negative rods frequently discovered in endodontic infections [28]. In addition to the categorization of bacteria, endodontic pathogenicity and bacterial resistance to antimicrobial treatments appear to be strongly influenced by the bacteria’s capacity to form biofilms [28].

The significantly higher total bacterial count observed when *Pseudomonas aeruginosa* was added directly after *Enterococcus faecalis*, followed by *Candida albicans* and *Proteus mirabilis*, may be attributed to a combination of specific microbial interactions and synergistic effects. This sequence of addition could create a particularly conducive environment for bacterial growth, potentially fostering synergistic interactions between *Pseudomonas aeruginosa*, *Enterococcus faecalis*, and subsequent species, as demonstrated in studies of mixed-species biofilms [1, 4]. Additionally, the competitive exclusion for nutrients and attachment sites among certain bacteria may have influenced biofilm composition, potentially contributing to the observed increase in bacterial count [9]. The timing of *Pseudomonas aeruginosa* introduction immediately after *Enterococcus faecalis* might establish a foundation for subsequent bacterial attachment and growth, thus influencing the overall biofilm structure and resulting in a higher total bacterial count. This specific order of addition could affect quorum sensing dynamics, a crucial coordination mechanism in biofilms, potentially further influencing biofilm development [4, 5]. These intricate interactions, coupled with potential metabolic exchanges and species-specific adhesion mechanisms, could collectively contribute to the observed increase in bacterial count [21, 27]. Finally, it is worth considering that the presence of certain bacteria early in biofilm formation may lead to changes in gene expression profiles of subsequent species, potentially influencing the overall biofilm structure and properties [6]. This highlights the multifaceted interplay of microbial interactions, competitive dynamics, quorum sensing, metabolic exchanges, environmental factors, adhesion mechanisms, and gene expression in biofilm development.

The genetic characteristics of the bacteria and the pellicle determine the adhesive interactions that cause a certain organism to adhere to it [1]. The first organisms to generate an endodontic biofilm are gram-positive cocci like *Enterococcus faecalis* [3, 4]. *Enterococcus faecalis* cells attach to the root canal dentine surface and grow into microcolonies, creating the endodontic biofilm [30]. After the mineral component of the dentine substrate is dissolved by bacteria, the biofilm begins to mineralize (or calcify), which results in a localized rise in the concentration of calcium and phosphate ions [30, 31]. The *Enterococcus faecalis* biofilm becomes mineralized as a result of the interaction between the bacteria and the metabolic products they produce with the dentine [18, 31]. *Enterococcus faecalis* and *Pseudomonas aeruginosa* co-aggregate, as shown by recent investigations. Their interactions during co-aggregation suggested that they may coexist in a microbial population and result in endodontic infection [28]. This interaction leads to the formation of a hostile environment for *Candida albicans* and *Proteus mirabilis*, which leads to a significantly greater optical density and bacterial count as shown in the second group [1, 31].

The present study has a limitation regarding the method used for detaching the biofilm from the substrate. The absence of visual confirmation through techniques like scanning electron microscopy (SEM) or confocal microscopy raises uncertainty about the effectiveness and consistency of the detachment process [1, 4]. Without visual evidence, it is difficult to assess if the method was applied uniformly across all groups, potentially introducing variability in the results [2, 3]. Additionally, the lack of imaging data hinders the ability to analyze biofilm structure and interactions between bacterial species [5]. Future studies could benefit from incorporating imaging techniques for a more reliable assessment of biofilm detachment.

## Conclusion

In this study, we observed a notable increase in total bacterial count in the group where *Pseudomonas aeruginosa* was added directly after *Enterococcus faecalis*, followed by *Candida albicans* and *Proteus mirabilis*, in comparison to the other groups. These findings shed light on specific aspects of endodontic biofilm development and offer insights into the dynamics of microbial interactions. Understanding these intricacies is pivotal for advancing the clinical success rates in endodontics. Further research in this direction will contribute to a deeper comprehension of bacterial traits, biofilm formation, and the alterations within the root canal environment, ultimately enhancing the efficacy of endodontic treatments.
